# Supporting Student Mental Health With the Safespace Generative AI Chatbot: Mixed Methods Feasibility Study

**DOI:** 10.2196/85427

**Published:** 2026-06-24

**Authors:** Matteo Pinna, Sergio Galletta, Elliott Ash, Timon Elmer

**Affiliations:** 1 Center for Law and Economics, Department of Humanities, Social and Political Sciences, ETH Zürich, Haldeneggsteig 4, Zürich, 8092, Switzerland, +41 44 6324586; 2 Safespace Research AG, Zürich, Switzerland; 3 Department of Economics and Law, Sapienza University of Rome, Rome, Italy; 4 Applied Social and Health Psychology, Department of Psychology, University of Zürich, Zürich, Switzerland

**Keywords:** generative artificial intelligence, GenAI, large language model, mental health, depression, chatbot, students, feasibility, mixed methods, qualitative content analysis

## Abstract

**Background:**

Generative artificial intelligence (GenAI) chatbots have the potential to provide personalized mental health support to individuals at scale.

**Objective:**

This study evaluates the feasibility and usage patterns of the Safespace GenAI chatbot, an artificial intelligence (AI)–driven smartphone app that offers a large language model–powered interactive chatbot to support mental health.

**Methods:**

Using a mixed methods approach, we explored baseline attitudes toward GenAI chatbots and chatbot usage patterns, conducted a qualitative content analysis of participants’ experiences, and descriptively assessed patterns related to preintervention depressive symptoms. The study included an initial sample of 42 university students, 20 of whom actively used the chatbot over 2 to 4 weeks, generating 286 user-chatbot interactions.

**Results:**

Preintervention surveys indicated that the majority of participants anticipated that the chatbot would be helpful (27/42, 64%) and that they trusted its privacy safeguards (39/42, 93%). Usage patterns suggested that the highest levels of interaction occurred early in the morning and late at night, when peer and professional support may be inaccessible. The qualitative analysis indicated that participants appreciated using the chatbot for reflection as a blended-care tool between their counseling sessions, while also naming technical barriers and specific design needs required to sustain engagement. In addition, our exploratory analyses descriptively showed that participants with elevated depression scores engaged in emotional disclosure during 99% (38 sessions with 8 participants) of their sessions, compared to 84% (26 sessions of 12 participants) of those with low symptoms. Due to the small sample size, future adequately powered studies are needed to inferentially examine these observed patterns.

**Conclusions:**

These findings provide initial insights into the usage and engagement dynamics of the Safespace GenAI chatbot and highlight directions for future research to optimize GenAI-driven mental health interventions.

## Introduction

### Potential of Generative AI Chatbots

The mental health crisis has intensified worldwide, with an estimated 60% of people with depression not receiving formal treatment [1]. In Switzerland, this challenge is compounded by a shortage of mental health workers and rising demand for mental health services [2], creating a need for interventions for help-seeking individuals [3]. These issues affect diverse populations, including university students, who face unique stressors such as academic pressure, new environments, and peer-related challenges. Indeed, studies have documented high rates of depression and anxiety among students, along with associated social and health consequences, emphasizing the need for tailored interventions [4-7].

In this context, eHealth tools like generative artificial intelligence (GenAI) chatbots offer promising solutions by providing accessible and personalized support to individuals [8], including those in academic settings. The use of artificial intelligence (AI) and chatbot technology, including advanced large language models (LLMs) like generative pretrained transformer (GPT), presents promising opportunities for mental health support. Studies have demonstrated their effectiveness in various applications, from offering empathetic conversations and mood monitoring to guiding peer-to-peer support and delivering psychotherapeutic programs [9-11].

This study aims to evaluate the feasibility of an LLM-based mental health chatbot through a formative mixed methods approach. Specifically, it seeks to provide insights into general temporal usage patterns, qualitatively explore the user experience and barriers to engagement, and descriptively assess usage patterns related to preintervention depressive symptoms.

A substantial body of research highlights the unique mental health pressures faced by the university student population [12]. A recent study of graduate students in European economics departments found that 34.7% experienced moderate-to-severe symptoms of depression or anxiety, with 17.3% reporting suicidal or self-harm ideation over a 2-week period, and only 19.2% of those affected were in treatment [4]. These striking figures complement earlier findings in the literature that report similarly high prevalence rates among university students across various disciplines. For instance, studies in the top US economics departments and broader academic settings have reported depression and anxiety rates ranging from 24.8% to over 40% [5]. Evans et al [13] surveyed 2279 graduate students across diverse fields and found that 41% (n=935) experienced moderate to severe anxiety and 39% (n=888) reported moderate to severe depression. Similarly, a meta-analysis by Satinsky et al [14] suggests that mental health challenges among PhD students are widespread, with depression and anxiety rates significantly exceeding those of the general population. Moreover, research suggests that access to mental health treatment remains limited, with many students either unaware of available resources or hesitant to seek professional help due to stigma or concerns over confidentiality [15,16]. Conversational rule-based (pre-LLM) chatbots have been shown to be useful in providing support for various mental health issues [17]. For example, Woebot delivered cognitive behavioral therapy through brief, daily conversations and mood monitoring and was found to reduce symptoms of depression in college students [9]. Another chatbot, Tess, was effective in providing psychological support for users with anxiety, depression, and stress [18]. Additionally, the chatbot Anna (Happify Health) uses natural language processing and machine learning algorithms to engage users in empathetic conversations and offer support for individuals at risk [19]. Other chatbots such as Replika [20] and Youper [21] further demonstrate the range of applications in this field. Although improvements in attrition and dropout rates have been observed compared with traditional digital interventions, attrition remains a significant concern. A potential reason is the limited guidance and support from peers or professional therapists in chatbot-based interventions, a factor shown to be important for other types of digital mental health interventions. Without such assistance, users may struggle to maintain engagement or effectively address their mental health needs, highlighting the potential of blended-care or stepped-care approaches. While these early systems show promise, their one-size-fits-all rule-based designs limit personalization, thereby paving the way for LLM-driven chatbots like Safespace, which can adapt conversation flow and memory to individual users.

GenAI models have been increasingly used in mental health applications due to their enhanced conversational capabilities. In particular, they have been explored in the context of cognitive behavioral therapy [22], highlighting their impressive language understanding and reasoning capabilities with potential applications in mental health care. One randomized controlled trial study using a GenAI chatbot for mental health showed that its use was associated with reduced depressive and anxiety symptoms [11].

### The Safespace AI Chatbot

The Safespace chatbot aims to counteract the attrition issues associated with earlier (non-LLM) chatbots [10], which typically offered a one-size-fits-all solution. In contrast, the Safespace chatbot becomes increasingly personalized as users engage with it. While previous work has shown the positive effects of rule-based chatbots on stress and anxiety, this feasibility study aims to expand the literature to the new generation of GenAI chatbots powered by LLMs. Compared to ChatGPT, Gemini, Claude, or other publicly available chatbots, Safespace focuses on user privacy and data security: user inputs are neither used to improve the language model nor accessible to researchers. These privacy measures are clearly presented to the users during the participation consent process. The Safespace chatbot allows users to engage in private, supportive conversations aimed at helping them cope with daily-life stressors.

### The Current Study

Since this is a feasibility study rather than a full-scale effectiveness trial, its primary focus is to examine the acceptability, user experience, and usage patterns of the Safespace chatbot. Specifically, the aims of this study are 3-fold: (1) to describe general app usage patterns, including temporal engagement (eg, time of day and week) and users’ preintervention attitudes toward AI; (2) to evaluate the user experience through a qualitative analysis of user feedback; and (3) to conduct exploratory quantitative analyses assessing how preintervention depressive symptoms are associated with chatbot engagement levels and the likelihood of emotional disclosure.

Understanding which user groups engage with the app, when they use it, and how they experience it—quantitatively and qualitatively—can inform future improvements for the Safespace chatbot and AI mental health tools in general. For instance, understanding specific barriers to engagement or how symptomatic students use the tool for emotional disclosure can help refine the chatbot interventions to better meet the needs of vulnerable populations in a blended-care context.

## Methods

### Study Design

This study used a mixed methods approach, collecting both quantitative app usage data and qualitative feedback through surveys and in-person follow-up sessions. Over a period of 2 months (starting in March 2024), participants from a university in Switzerland were invited to use the app, including the chatbot, as part of a naturalistic trial, with data continuously logged during the intervention period. Students were recruited through a local student counseling office. They attended counseling either for personal or mental health–related concerns or for career advice. The selection of participants was based on the judgment of the counselors, and students deemed to be at high mental health risk were not offered the app. Additionally, students who scored above 20 on the Patient Health Questionnaire-9 (PHQ-9) depression scale [23] or above 0 on the PHQ-9 self-harm item were excluded from participation. A total of 100 students were offered to participate in the study; 47 accepted, 42 completed the survey, 20 had sufficient chatbot data for analysis (at least 1 user-chatbot interaction marked as opening up, with 15/20 or 75% of users having more than 3 user-chatbot interactions and 10/20 or 50% having more than 6.5 interactions), 6 completed the follow-up survey, and 22 provided qualitative feedback (3 through the follow-up survey and 19 through postintervention sessions with counselors). The sample consisted of two-thirds Swiss students, with an average age of 24 years (see Figures S1 and S3 in Multimedia Appendix 1). Gender distribution was 48% (20/42) male participants, 43% (18/42) female participants, 4.5% (2/42) nonbinary, and 4.5% preferred not to share (2/42; see Figure S2 in Multimedia Appendix 1). The surveys and randomization were implemented through Qualtrics, with two-thirds of the participants receiving the app for 4 weeks and one-third for 2 weeks. The goal of the randomization was to assess the effects of the intensity of the intervention, defined as the availability of the chatbot. However, because of the too-low sample size in the 2-week treatment group, we could not offer meaningful insights about the heterogeneity in usage or postintervention outcomes between the 2 experimental groups.

### Prepilot Tests

Initial feedback on the chatbot and survey was gathered through an internal testing within the research team, involving 15 participants. The application was then provided to counselors at the partner university for further testing and feedback, with 6 counselors testing the app. Their most valuable input focused on refining the suicide-risk response, which was partially rewritten and adjusted. Additionally, their feedback led to adjustments in the chatbot’s tone, making responses more balanced in terms of text length. To further evaluate chatbot interactions, we scraped 100 Reddit posts from mental health subreddits and used them to simulate user queries. This process revealed 2 main issues: some chatbot responses contained gibberish text, while others hinted at diagnostic language. These insights led to the implementation of quality control measures to filter gibberish responses and the addition of explicit safeguards against clinical diagnostic discussions.

### Procedure and Preregistration

Participants were recruited through a Swiss university’s counseling services and provided informed consent prior to participation. After providing consent, participants completed a baseline survey to assess their demographic characteristics, mental health–related constructs (eg, anxiety symptoms and depressive symptoms), and AI-related beliefs, building on previous research by Macchi et al [4]. A unique identifier was provided at the end of the preintervention survey, which the participants could use to log in to the Safespace app. No email addresses were collected, and researchers had no means to contact the participants, as outlined in the approved ethics proposal. They then used the Safespace app for either 2 or 4 weeks, during which app usage data were automatically recorded, excluding the content of the interactions (which is exclusively saved in the memory system to which the researchers did not have access). At the end of the study period, participants were invited to complete an end-of-study survey. Additionally, a subset of participants took part in an in-person voluntary follow-up session, where qualitative feedback regarding their experiences and the app’s impact on their mental health was collected through a qualitative interview.

### The Safespace AI Chatbot

**Figure 1.**
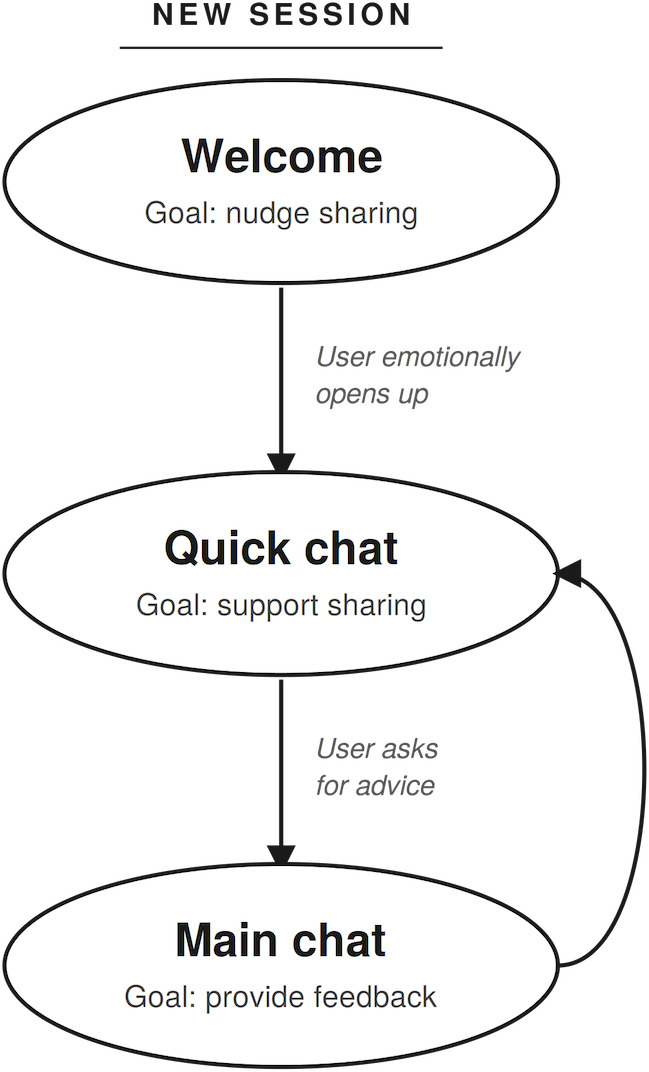
Basic structure of Safespace’s conversational state machine.

Safespace is structured as a state machine, where a system of text-based classifiers determines the user’s state, such as whether they are emotionally opening up, as expressive disclosure of negative emotions can have mental health benefits [24]. The AI adapts its behavior accordingly, while an auxiliary AI continuously monitors the conversation to assess whether a state transition is necessary. The main conversational states capture whether the user has emotionally opened up to the chatbot and whether the user is requesting advice (the basic structure of a conversation’s state is shown in Figure 1). During the pilot study, the model configuration was fixed: GPT-4‐0314 was used for the main conversational output, while GPT-3.5-turbo-0125 handled length adjustments and GenAI-aware concatenation of outputs—either between the 2 models or across prompt variations within a single model. Additional personalization of chatbot responses was enabled by its memory system, which retained relevant user interactions, similar to the notes-taking process of a psychotherapist. This system operated in 2 steps: first, conversations were summarized into paragraphs, and key details were stored as short entries. These key details were categorized into sensitive (eg, relationships, family, and health-related topics) and nonsensitive (eg, hobbies and sports) information. Together with real-time conversation summaries, these constituted memory layers of Safespace [25]. Before storage, information was timestamped, and repeated entries were removed to prevent noise accumulation. For this feasibility study, the memory layers were stored locally on the user’s device and compiled over time. The second step of the memory system was designed for follow-up studies with longer usage periods beyond the current 2- to 4-week feasibility study. LLMs are known to exhibit attention issues when handling long contexts, often assigning importance based on text location rather than relevance [26,27]. To address this, Safespace stores information in a structured database and retrieves only contextually relevant data for each user interaction. The retrieval process considers multiple factors, including relevance and time weighting, to prioritize the most useful information.

Safespace limits the scope of conversations to mental health–related topics through a combination of prompt engineering and text-based classification. Students were restricted from asking for direct academic assistance (except for topics such as stress management or study-structure advice) and could not request dietary or medical recommendations. Prepilot testing using Safespace chatbot interactions with Reddit-scraped data revealed a tendency for users to seek self-diagnoses, leading to a safeguard against diagnostic discussions (see the Methods section). However, these restrictions were not applied in an overly rigid manner. For example, the chatbot may provide general guidance on low-risk health-related queries (eg, “Is taking 600 mg of ibuprofen safe?") while still advising users to consult a medical professional.

Additionally, in cases where self-harm or suicidal ideation was detected, the chatbot immediately suspended LLM-generated responses and provided a tailored emergency intervention message designed in collaboration with university counselors. This detection is achieved through an auxiliary AI model that continuously monitored conversations and classified risk levels to determine when an intervention was necessary. It then presented the user with quick access to emergency mental health resources, including helplines and crisis support services, along with information on their availability.

The Safespace chatbot integrates multiple machine learning technologies, including summarization of sensitive content, information retrieval, and language models. The state-machine framework supports various LLM models, and several providers were considered in the design stage: OpenAI, Anthropic, Gemini, and Llama. The final configuration for the feasibility study consisted of a hybrid approach, using a combination of GPT-3.5-turbo, GPT-4 (March 13 version), GPT-4o-mini, and semantic similarity models from OpenAI. Heavier models handled the main chatbot interactions (GPT-4-0314), while lighter models were used for summarization and state-machine classification (GPT-3.5-turbo-0125). These models were judged by the researchers to be the best-performing and safest models for the task at the time of the pilot study.

Currently, the Safespace chatbot is text-based and does not support voice-to-voice interaction. However, a text-to-speech and speech-to-text feature was included in the initial implementation. User feedback indicated low usage of these features. Qualitative discussions with university counselors suggested that users may prefer interacting with the chatbot in private settings where speaking aloud is not feasible, such as during quiet moments of reflection or when privacy concerns arise.

### Measures

The survey included demographic questions covering age, gender, nationality, language proficiency, relationship status, and living situation, along with educational background, including student status and field of study (for details, see Multimedia Appendix 1). Additionally, participants reported whether they had been referred to the study by university counseling services.

Mental health measures included standardized scales for depression (PHQ-9 [23]) and anxiety symptoms (General Anxiety Disorder-7; GAD-7 [28]). Given ethical considerations, the self-harm question from the PHQ-9 was modified at the request of the Ethics Committee. The original wording, “Thoughts that you would be better off dead or of hurting yourself,” was adjusted to “Thoughts of not wanting to be alive or causing harm to oneself” to reduce the risk of triggering distress in participants. In addition to mental health measures, the survey collected attitudes and expectations regarding AI-driven mental health support. Participants rated their trust in AI, perceived usefulness of chatbots for mental health, and expectations regarding privacy protection within the Safespace app (for details, see Section S3 in Multimedia Appendix 1).

The follow-up survey, administered after participants had used the Safespace chatbot for 2 to 4 weeks, aimed to assess app engagement, user experience, and changes in mental health measures (for details, see Section S4 in Multimedia Appendix 1). Participants reported how frequently they used the app for emotional or mental health–related support and whether they felt comfortable opening up to the chatbot. Additional items assessed perceived effectiveness, ease of use, and trust in the app’s privacy features. In addition to the information collected through surveys, we gathered app usage data to analyze engagement patterns. The recorded metrics included the number of accesses, total time spent on the app, and user interactions with various app components, such as clicks on emergency resources and mood-tracking features. Regarding chatbot interactions, we collected data on the number of interactions, the length of user input and chatbot output, and the chatbot’s state-machine classification at each interaction. This allowed us to determine whether a user emotionally opened up to the chatbot. However, we did not have access to the chat content or the memory system of the app. Participants were informed about data collection procedures in the consent form.

To determine whether a person emotionally opened up, we analyzed the output of one of the state-machine classifiers, designed to detect whether a user emotionally opens up to the chatbot. The emotional-openness classifier was implemented using a GPT-3.5-turbo model, with the LLM serving as a judge [29]. The classifier used a prompt that explicitly asks about emotional openness, with answer-type restrictions: “In the conversation above between a user and an AI chatbot, is the user emotionally opening up to the chatbot?” This prompt was validated on a test dataset and outperformed several descriptive and example-based alternatives. Recent work (Pinna et al, unpublished data, 2026, working title: Measuring emotional openness and adherence with LLMs: capturing components of therapeutic alliance in mental health chatbots) builds on this by fine-tuning a more recent medium language model on a dataset of approximately 100 real mental health user messages from Reddit, annotated by ~120 psychology experts, collected to study pluralistic alignment. When evaluated post hoc on the data from the above mentioned unpublished study, the GPT-3.5-turbo model prompt achieved 95% agreement with human labels, treating a message as “user emotionally opening up” whenever at least 1 expert judged it as such. To quantify the frequency of emotional openness, we defined an indicator variable for sessions containing at least 1 emotional opening-up interaction. The classifier operates only during the initial phases of each new conversation to determine when to transition from an ice-breaker style to a journaling-focused conversational mode that encourages emotional disclosure. A session was defined as a sequence of chatbot interactions separated by at least 2 hours of inactivity. A chatbot interaction was defined as the user sending at least 1 message and receiving a chatbot response.

### Qualitative Content Analysis

To evaluate the user experience and acceptability of the Safespace application, we conducted an inductive qualitative content analysis [30] on the collected user feedback. Qualitative data were gathered from 2 distinct sources: open-ended responses in the postintervention survey (3/20, 15%) and transcribed notes from follow-up interviews conducted by university counselors (19/20, 95%). The data were systematically reviewed, and initial codes were generated by a single researcher. These codes were iteratively updated as the data were coded. These codes were subsequently grouped into categories.

### Exploratory Statistical Analyses

Descriptive statistics were computed for all demographic, mental health, and usage variables, and group comparisons (eg, low vs mild-to-moderate depression scores) were conducted. Given the nature of this study and the small sample size, these analyses were intended to be exploratory and descriptive. Hence, no inferential statistical tests were conducted.

### Ethical Considerations

The study protocol, including all data collection procedures and privacy safeguards, was approved by the ethics committee of ETH Zurich (EK-2023-N-184) and preregistered at the AEA Registry. Written informed consent was obtained from all participants prior to their participation in the study. To guarantee complete participant anonymity, no contact details (such as email addresses) were collected, ensuring researchers had no means to identify or recontact participants. Furthermore, strict data privacy measures were implemented: the content of the user-chatbot interactions was exclusively saved in the app’s memory system, and researchers did not have access to the raw chat logs. Participants were fully informed about these data collection procedures in the consent form.

## Results

### Preintervention Attitudes

**Figure 2.**
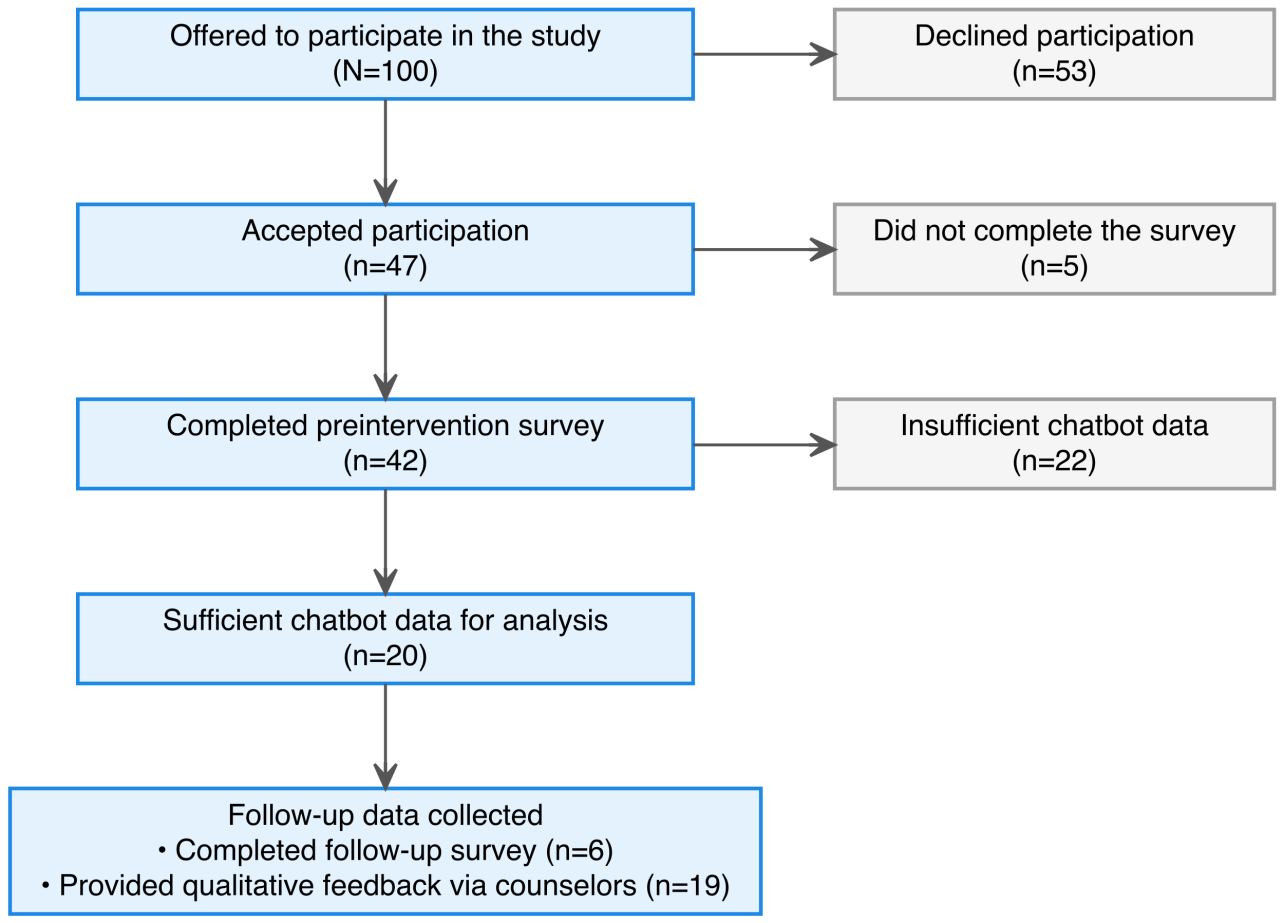
Flow diagram of the study.

Figure 2 shows a flow diagram of the participants at the various points of data collection. Participants’ responses before the intervention to AI-related questions suggested positive attitudes, with 64% (27/42) believing the chatbot would be helpful, compared to 21% (9/42) who were skeptical (Figure S6 in Multimedia Appendix 1). Similarly, 93% (39/42) of users reported feeling their privacy would be safeguarded, while only 2% (1/42) expressed concerns (ie, “somewhat disagree” (4) on a 5-point scale; Figure S7 in Multimedia Appendix 1).

### App Usage Patterns

**Figure 3.**
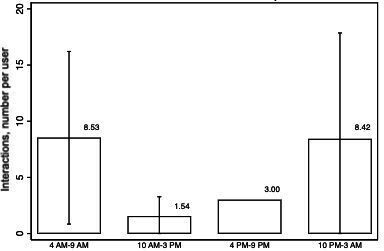
Number of average chat interactions by time of day per user. Error bars are 95% CI; the 4 PM to 9 PM group contains a single user, and so no interval is shown. Most interactions take place early in the morning or late at night.

Participants engaged with the chatbot an average of 5 times (SD 5.55). The average number of sessions per user was 2.85 (SD 2.62), and the weekly in-app screen time was 9.34 (SD 10.7 min). A user had an average session of 86 (SD 102) tokens, corresponding to approximately 40 to 60 user-input words (a token is the basic unit of text an AI model processes, typically 3 to 4 characters or about three-fourths of a word). Despite the distribution being skewed, the overall average session had 62 (SD 106) tokens, showing that users with a high number of sessions tended to write shorter messages. Usage peaked on Fridays and Saturdays, with activity levels being 50% higher and nearly double compared to other days (see Figure S8 in Multimedia Appendix 1). In addition, students were more likely to use the chatbot late at night or early in the morning as, on average, students had 17.53 (SD 18.17) interactions with the chatbot between 10 PM and 9 AM, compared to only 4.60 (SD 1.29) interactions on average during the rest of the day (Figure 3).

### Qualitative Analyses

These qualitative feedback reports, obtained after the intervention period, are presented in detail in Section S2 in Multimedia Appendix 1 (including English translations from the original German). Based on these data, 32 unique codes and 4 categories were derived. Given the small number of categories, we decided not to derive further overarching themes or main categories [31]. The codes and categories are listed in detail in Multimedia Appendix 2.

The first category, “Blended Care and Reflection Utility,” captures how participants used the chatbot as a helpful blended-care tool. Participants frequently referred to the chatbot as a *useful addition to human counseling* (n=4) and reported an overall *positive use* (n=4) of the chatbot. One person reported that they “[...] *would like to continue using the app. It feels like chatting with a real person, but I don’t see it as a replacement for coaching—rather, it’s a good in-between option*.” Rather than replacing human interaction, the chatbot served as a dedicated space for reflection between the counseling sessions. Similarly, a participant noted regarding their reflection and blended-care experience:

*I used it to write down my thoughts and put them into words. It was very helpful to receive responses that either confirmed my thinking or provided a suggestion for reflection. I can imagine that using the app alongside coaching could be useful.*

The second category, “Perceptions of AI Interaction,” entails mixed feedback regarding the chatbot’s conversational depth. On the positive side, users appreciated receiving *comprehensive answers* (n=2), alongside *solution-focused* (n=1) and *empathic* (n=1) responses. On the other hand, participants identified limitations, most notably a *lack of conversation depth* (n=2), such as “[...] it would be better if it provided more detailed responses and asked follow-up questions—so that it feels more like a conversation rather than a back-and-forth Q&A.”

The third category, “Barriers to Usage,” entailed some practical hurdles that hindered continuous engagement. The most prominent barrier by far was *technical issues* (n=5), which included a multistep installation process (eg, through TestFlight) and in-app bugs or formatting issues. Beyond technical friction, participants cited a *preference for human contact* (n=2) and frequently reported simply *forgetting about the chatbot option* (n=2). Additionally, some participants noted that *opening up was difficult* with an AI chatbot (n=1). For example, “*For me, it felt weird opening up to a chatbot. I much rather would talk to a friend face-to-face, but that’s just my personal preference.*

Finally, the fourth category, “App Design Needs and Engagement,” entailed participant suggestions for future iterations to overcome these barriers. To promote long-term use, the most frequent requests were *feature wishes for push notifications* (n=3) and *uplifting messages* (n=2). Participants suggested that automated, encouraging reminders would effectively prompt them to interact and prevent them from forgetting about the chatbot. Furthermore, participants noted that the app was generally *easy to use* and explicitly stated that having the *emergency contact* permanently visible was a highly useful safety feature.

### Exploratory Quantitative Analyses on Depressive Symptoms and Usage Patterns

**Figure 4.**
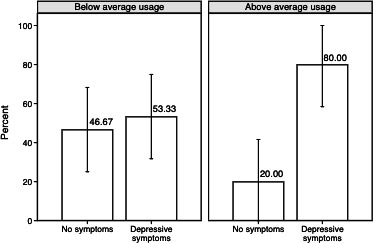
Above-average number of interactions between the participant and the chatbot by depression symptoms. The histogram descriptively shows a higher prevalence of depressive symptoms, with at least mild symptoms in participants with higher usage.

**Figure 5.**
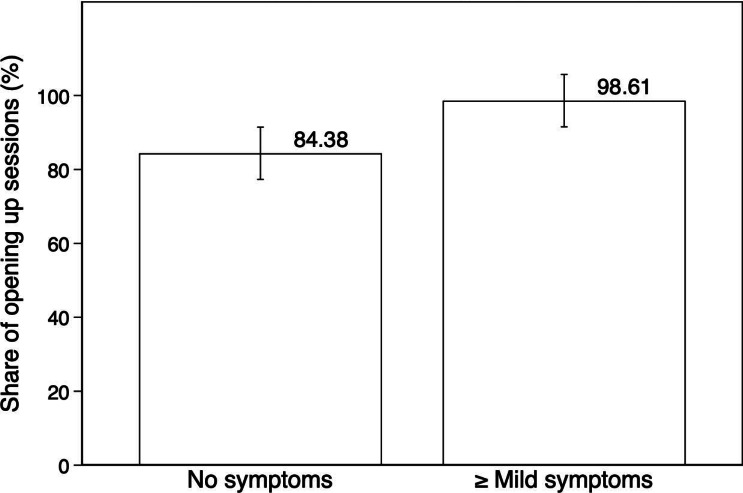
Share of sessions with at least 1 opening-up interaction categorized by preintervention depression scores. Notes: The histogram shows a 15% difference in the share of opening-up sessions between people with low (PHQ-9<5) and mild (PHQ-9=5‐9) depression symptoms, and a t test comparing the mean share of opening up between the 2 depression severity groups (t18=−2.11, P=.049).

Among the 20 students who used the chatbot and were included in the analysis, 8 (40%) exhibited low symptoms of depression (PHQ-9<5) and 9 (45%) of mild symptoms (4<PHQ-9<10), while 3 (15%) students had moderate symptoms (PHQ-9>10) according to the criteria by Kroenke et al [23]. We further divided the sample into below-average and above-average users (the average was 4.7, SD 5.55 weekly interactions with the chatbot). As shown in Figure 4, descriptively, there was a nearly 30% difference in the share of students with mild symptoms (n=4, 20%) and low symptoms (n=1, 5%), compared to the same difference in the lower chatbot engagement sample (n=8, 40% and n=7, 35%, respectively), indicating that those with mild or moderate levels of depressive symptoms might be more likely to be in the above-average user group than those with low symptoms of depression. Figure 5 shows the share of sessions in which participants emotionally opened up by depressive symptom group (no and low depressive symptoms vs mild and moderate depressive symptoms). Figure 5 also indicates an approximately 15% difference in the number of sessions involving emotional disclosure in the group with at least mild (PHQ-9=5‐9) depressive symptoms, compared to the group with low (PHQ-9<5) depressive symptoms. A 2-tailed *t* test was conducted comparing the mean share of opening up between the 2 depression severity groups: *t*_18_=−2.11, *P*=.049.

## Discussion

### User Experience and Engagement Patterns

This study aimed to examine the user experience and engagement patterns of a GenAI mental health chatbot called Safespace among university students. Specifically, we explored general app usage patterns, conducted a qualitative content analysis of user feedback, and descriptively assessed how preintervention depressive symptoms were related to chatbot engagement and emotional disclosure.

Our findings yielded 4 main insights into how students used the Safespace chatbot. First, regarding general acceptability, baseline responses indicated a willingness to engage with AI-driven support: 64% (27/42) of students anticipated the tool would be helpful, and 93% (39/42) trusted its privacy safeguards. These findings align with the existing literature highlighting the general acceptability and perceived helpfulness of mental health chatbots [21,32]. Notably, the high level of privacy trust observed here contrasts with the skepticism typically directed toward general-purpose and commercial applications [33,34]. This elevated trust may be attributable to Safespace’s university affiliation and the transparent communication of its ethical standards (eg, not collecting conversation data).

Second, regarding general usage patterns, we found that the overall interaction volume was disproportionately concentrated on weekends and between 10 PM and 9 AM, suggesting that students turn to the chatbot during late-night or early-morning hours. Together, these patterns hint that more symptomatic students not only use the chatbot more but also disclose more emotionally, especially during off-peak hours. This pattern highlights the potential of AI-driven interventions to fill critical gaps in mental health care availability. While general smartphone usage among students is known to peak during mornings and late nights [35], previous studies on mental health chatbots in the general population have conversely observed higher engagement during working hours [32]. Our results contrast with this “working-hours” pattern, showing that students turned to the Safespace chatbot primarily during late-night and early-morning intervals. This suggests that, for students, the chatbot serves a distinct function by filling a critical gap in care when professional services and peer support are typically inaccessible. However, further inferential analyses with larger samples are needed to substantiate these findings.

Third, our qualitative content analysis suggests that participants valued the Safespace chatbot as a supplementary blended-care tool, using it to journal, organize thoughts, and reflect on topics between professional counseling sessions. This supports previous research emphasizing that digital mental health interventions are often most effective and show lower attrition when deployed as adjuncts to human-guided counseling rather than standalone solutions [36]. GenAI chatbots have the potential to bridge the time between appointments by facilitating reflection, provided they are deployed within a clear framework that does not undermine or replace the human therapeutic relationships [37]. Furthermore, participants positively perceived the AI chatbot’s empathic and constructive tone, mirroring recent studies showing that LLMs can generate highly empathetic responses [38,39]. At the same time, they also recognized its limitations, such as a lack of conversational depth. This is in line with existing literature stating that while GenAI can successfully facilitate emotional disclosure and therapeutic alliance [11,40,41], it cannot replicate a deeper level of exploring cognitive and emotional processes that happen during face-to-face human counseling [42]. Furthermore, it must be emphasized that conducting formal psychotherapy should not be the aim of GenAI chatbots [43], as significant safety and quality concerns remain (eg, regarding a confrontational role that human therapists may take up) [44]. Finally, the barriers identified by participants, including technical friction, adherence to regular mood tracking, and privacy concerns, echo well-documented challenges in digital mental health [33]. Their requests for push notifications underscore the ongoing need to integrate reminders or even adaptive intervention timing [3,45] to overcome high attrition rates in mental health apps [8].

Fourth, exploratory quantitative analyses descriptively indicated that students with elevated depressive symptoms not only engaged more frequently with the chatbot but were also more likely to emotionally open up during their sessions compared to those with low symptoms (99% vs 84% of participants). Due to the small sample size, no inferential tests are reported, and we therefore do not interpret these descriptive quantitative tendencies further. However, these preliminary patterns highlight critical avenues for future, adequately powered research. Specifically, future studies could investigate whether frequent chatbot engagement among symptomatic students reflects adaptive help-seeking [46] or rather a form of “AI chatbot dependence” [47]. Unlike the maladaptive or compulsive usage patterns described in the context of general-purpose or companion GenAI chatbots [34,48-52], the usage observed here (averaging low weekly chatbot use) does not reflect a problematic dependence. Hence, researchers stress the need to differentiate between general-purpose and specific AI applications [53]. Overall, however, these findings show that more research is urgently needed to identify when an AI chatbot is adaptive and when it is maladaptive or even considered unsafe [34,48-51,54,55].

Furthermore, future research should rigorously test whether GenAI chatbots can lower barriers to emotional disclosure. In traditional counseling psychology, depressive symptoms are typically associated with reduced disclosure and increased avoidance [56]. If larger trials confirm that symptomatic individuals are more willing to disclose distress to an AI than to a human clinician due to stigma, chatbots like Safespace could be formally positioned as an effective “bridge” to professional care. Providing a safe, low-barrier environment to practice vulnerability could “warm up” users for more effective engagement with human consulting [57].

It is important to consider a few limitations of this study. First, the findings presented are based on a limited sample size and should be interpreted as exploratory rather than conclusive. Given the small sample size, the analyses are interpreted as descriptive patterns rather than inferential findings and warrant further statistical testing in larger samples. While the observed data describe how students interact with the chatbot, further data collection and analysis are necessary. A larger data collection and a randomized trial are needed to determine whether chatbot engagement leads to meaningful psychological benefits over time.

Second, this study specifically examined students who were attending counseling, a group that may be inherently more motivated to work on self-improvement and self-reflection. As a result, their engagement with the chatbot may not be representative of the general student population. Additionally, the higher usage rates observed could be influenced by the study setup itself, as students were encouraged to use the chatbot between counseling sessions as a form of supplementary support.

Another limitation of the study was specifically the lack of follow-up survey responses. As emails were not collected purposefully to guarantee anonymity, we could not reach out to the participants and send them reminders. This restricted our ability to conduct follow-up assessments, highlighting an area for improvement in future research designs.

### Conclusions

Our findings provide initial mixed methods evidence on the feasibility, user experience, and engagement dynamics of the Safespace GenAI chatbot. Overall, we found that participants anticipated the chatbot would be helpful and held strong trust in its privacy safeguards. Chatbot usage was heavily concentrated during weekends and late-night and early-morning hours, suggesting that the chatbot may fill a critical “care gap” when professional and peer support are typically inaccessible. Qualitative analyses further highlighted the chatbot’s promising utility as a supplementary “blended-care” tool for journaling and reflection between counseling sessions. Together, these patterns indicate that GenAI tools have the potential to provide an accessible, low-barrier space for self-reflection and emotional disclosure precisely when traditional support is unavailable. However, given the exploratory nature and small sample size of this study, well-powered studies are needed to rigorously test these trends and the efficacy of the chatbot to support students’ mental health.

## Supplementary material

10.2196/85427 Multimedia Appendix 1 Additional analyses, user feedback, and preintervention and postintervention survey materials related to the Safespace chatbot.

10.2196/85427 Multimedia Appendix 2 Coding report of qualitative analysis.

## Abbreviations

AI: artificial intelligence
GenAI: generative artificial intelligence
GPT: generative pretrained transformers
LLM: large language model
