# Supplementary material for: Supporting Student Mental Health With the Safespace Generative AI Chatbot: Mixed Methods Feasibility Study
Source: JMIR Form Res. 2026 Jun 24;10:e85427. doi: 10.2196/85427 (PMC13293566; doi:10.2196/85427)
Supplement: Multimedia Appendix 1 [file formative-v10-e85427-s001.pdf]

Supplementary Materials

Contents

S.1 Additional Analysis 2
S.1.1 Demographics and Survey Elements . . . 2
S.1.2 Chatbot Usage and Mental Health . . . 6
S.1.3 Comparison Between Chatbot Users and Non-Users . . . 10
S.1.4 Anxiety Measures . . . 11

S.2 User Feedback on the Safespace App 13
S.2.1 Feedback from Qualtrics Surveys . . . 13
S.2.2 Feedback from Follow-Up Interviews with University Counselors . . . 13

S.3 Survey Pre-Intervention 15

S.4 Survey Post-Intervention 23

S.1. Additional Analysis

S.1.1. Demographics and Survey Elements

Figure S.1 presents the share of Swiss students in the sample. Given the study was conducted in a Swiss university, the majority of participants are Swiss.

Figure S.1: Share of Swiss Students

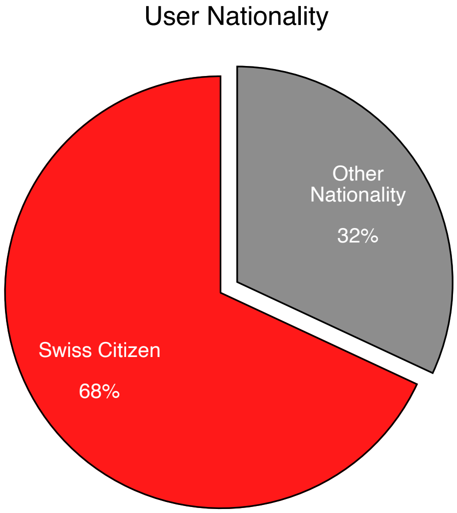

Figure S.2 shows the gender distribution of the sample.

Figure S.2: Gender

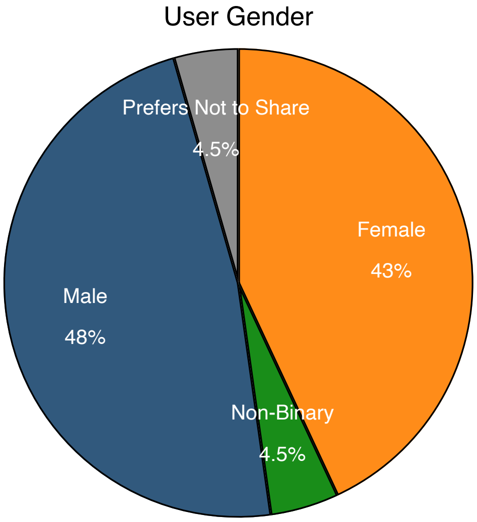

Figure S.3 displays the age distribution of participants. The results are consistent with expectations for a university student population, with most respondents falling in their early to mid-twenties.

Figure S.3: Age

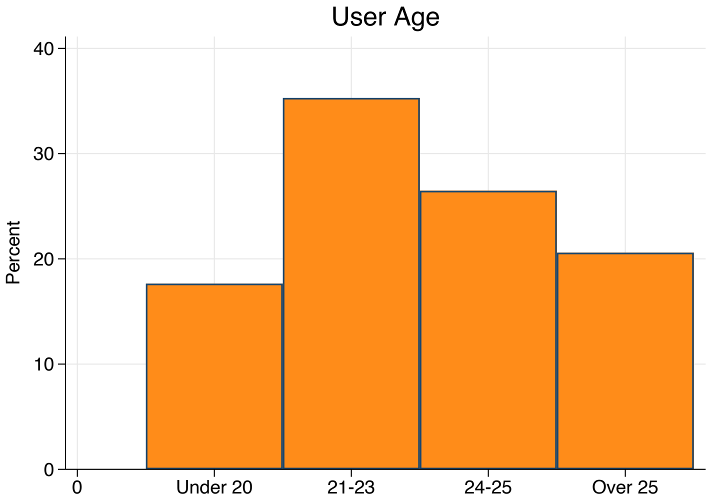

Figure S.4 reports the percentage of students living alone. It is common in Switzerland for students to share accommodations in WGs (Wohngemeinschaften) to split living costs, making this distribution reasonable.

Figure S.4: Living alone status

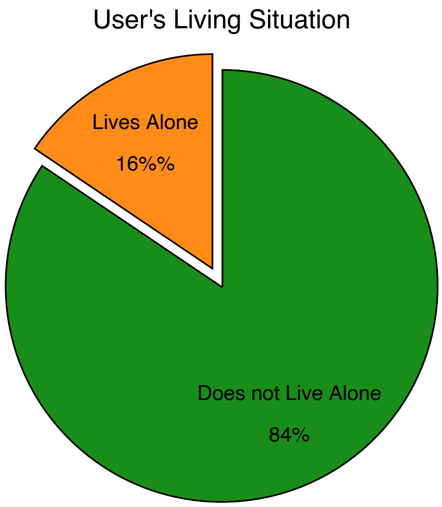

Figure S.5 highlights that 98% of participants reported having at least one person to emotionally confide in, indicating strong levels of perceived social support.

Figure S.5: Number of people users feel they can emotionally open up to

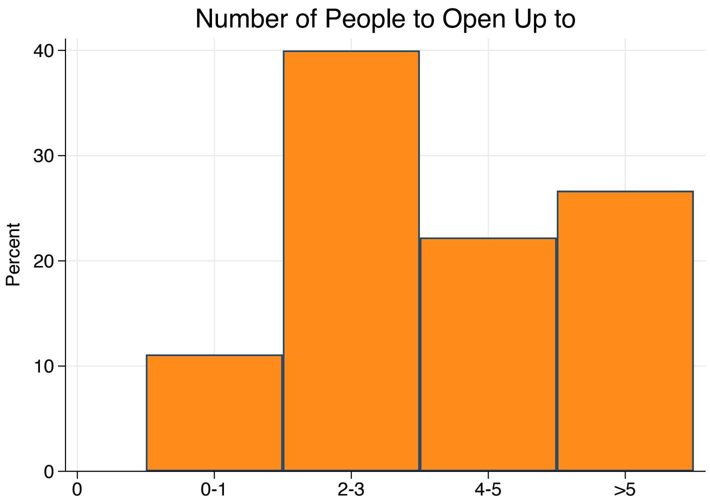

Figures S.6 and S.7 show participants' attitudes toward AI-based mental health tools. A **majority (64%) believed the chatbot would be helpful**, while **93% expressed trust in the app's privacy protection**.

Figure S.6: User perceptions: AI helpfulness.

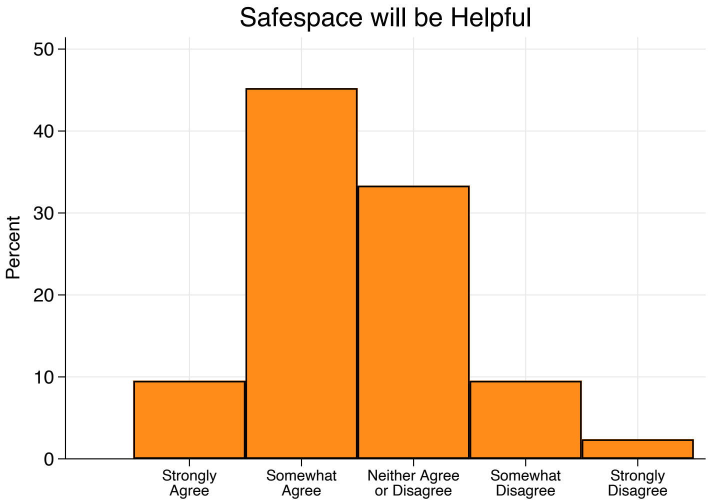

Figure S.7: User perceptions: Privacy protection.

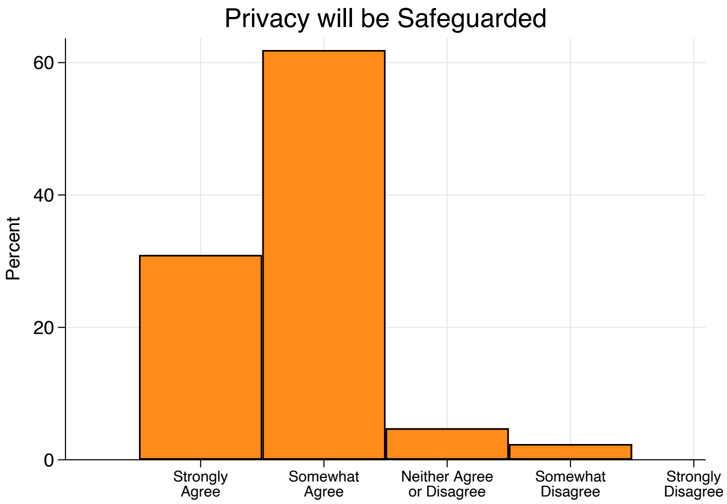

S.1.2. Chatbot Usage and Mental Health

Figure S.8 shows weekly chatbot interactions across different days. The x-axis represents the number of chatbot interactions per week.

Figure S.8: Chatbot interactions across different days

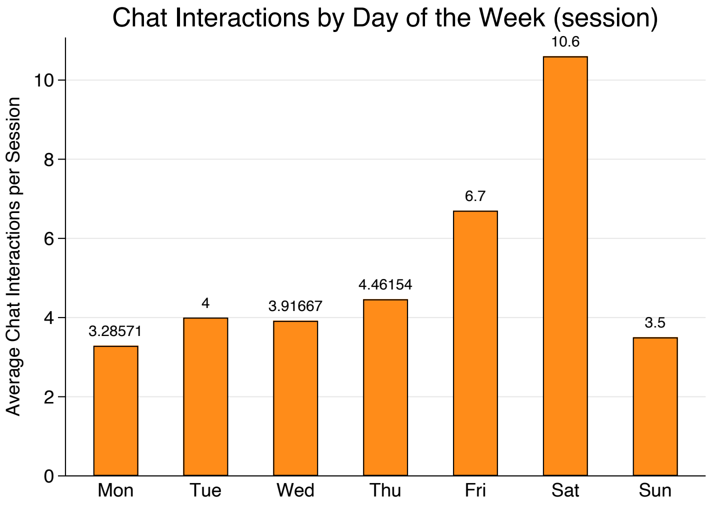

Figures S.9 and S.10 explore the relationship between chatbot usage and PHQ-9 depression scores for students living alone. The PHQ-9 scale categorizes depression severity as: 1 = Mild symptoms, 2 = Moderate symptoms, 3 = Moderately severe symptoms, 4 = Severe symptoms.

Figure S.9: Chatbot usage by living alone status

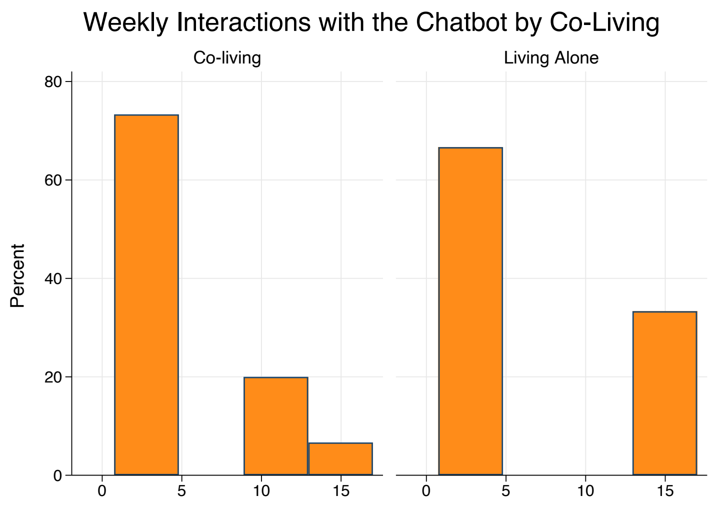

Figure S.10: PHQ-9 depression scores by living alone status

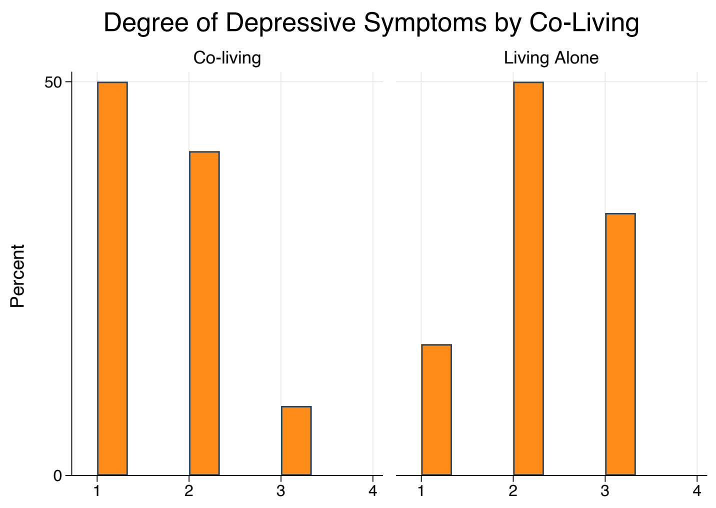

Figures S.11 and S.12 explore the relationship between chatbot usage and PHQ-9 depression scores for **Swiss vs. non-Swiss students**. The PHQ-9 scale categorizes depression severity as: 1 = Mild symptoms, 2 = Moderate symptoms, 3 = Moderately severe symptoms, 4 = Severe symptoms.

Figure S.11: Chatbot usage by Swiss vs. non-Swiss students

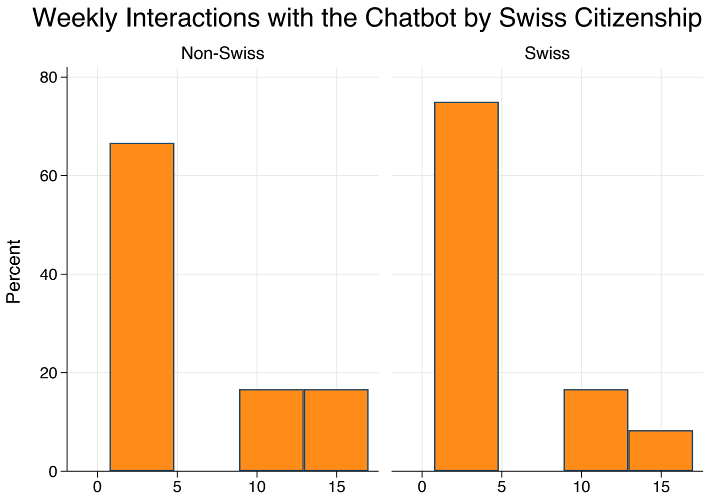

Figure S.12: PHQ-9 depression scores by Swiss vs. non-Swiss students

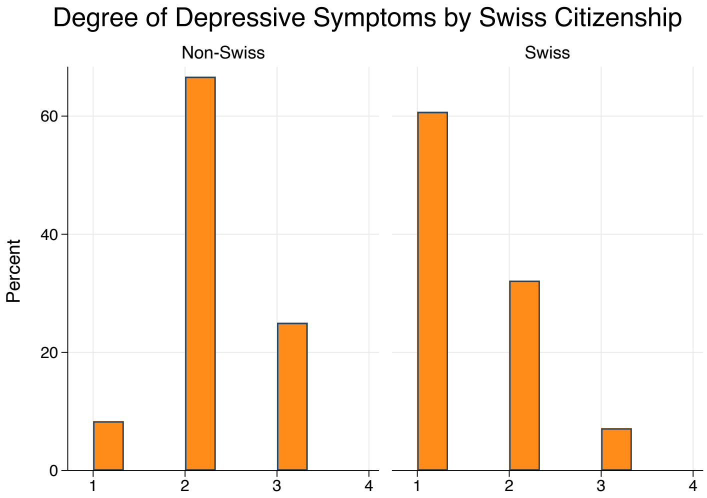

Figures S.13 and S.14 examine **gender differences in chatbot usage and PHQ-9 depression scores**. The PHQ-9 scale categorizes depression severity as: 1 = Mild symptoms, 2 = Moderate symptoms, 3 = Moderately severe symptoms, 4 = Severe symptoms.

Figure S.13: Chatbot usage by gender (male vs. female/non-binary)

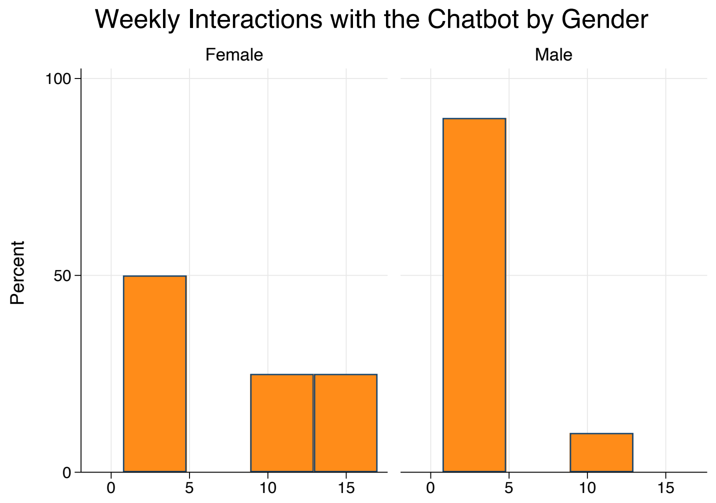

Figure S.14: PHQ-9 depression scores by gender (male vs. female/non-binary)

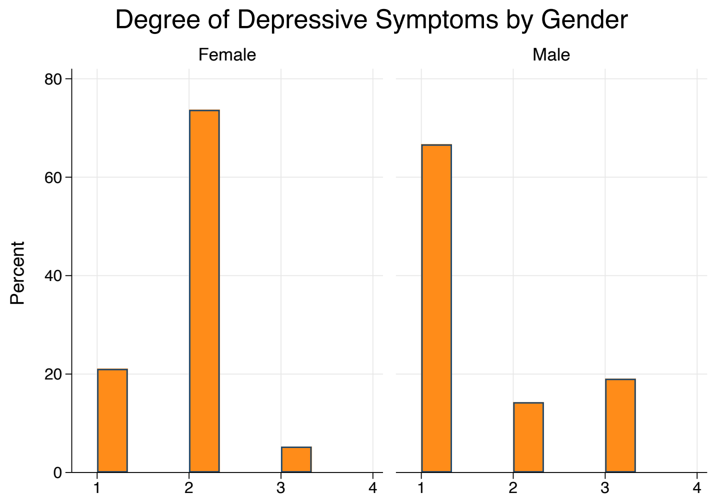

S.1.3. Comparison Between Chatbot Users and Non-Users

Figures S.15 and S.16 compare PHQ-9 depression levels and anxiety levels between chatbot users and non-users. In both cases, 0 represents no symptoms, and 1 represents mild symptoms.

Figure S.15: PHQ-9 depression levels for chatbot users vs. non-users.

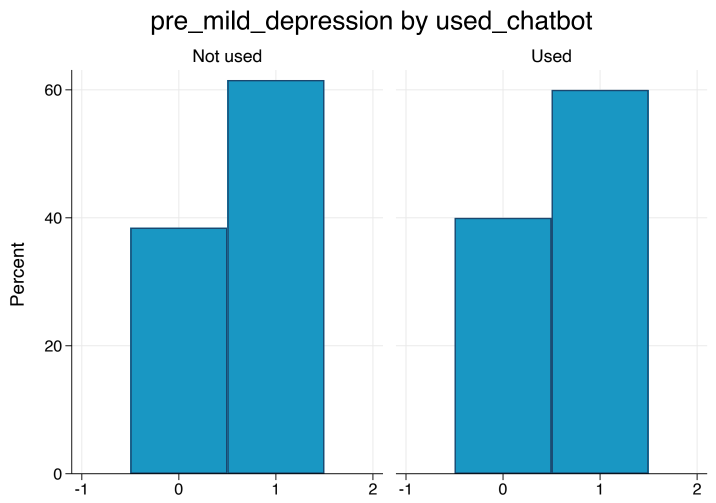

Figure S.16: Anxiety levels for chatbot users vs. non-users.

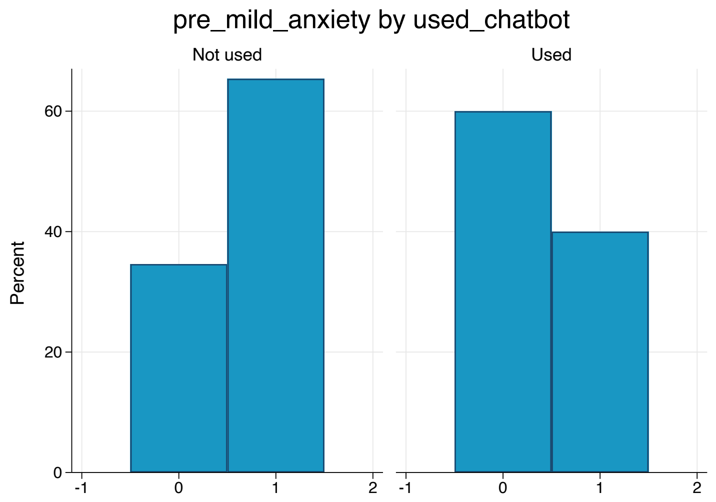

S.1.4. Anxiety Measures

Figures S.17 and S.18 show above average number of interactions between the subject and the chatbot, by anxiety symptoms, and the difference in the share of opening up sessions, by anxiety symptoms.

Figure S.17: Anxiety levels by weekly chatbot interactions.

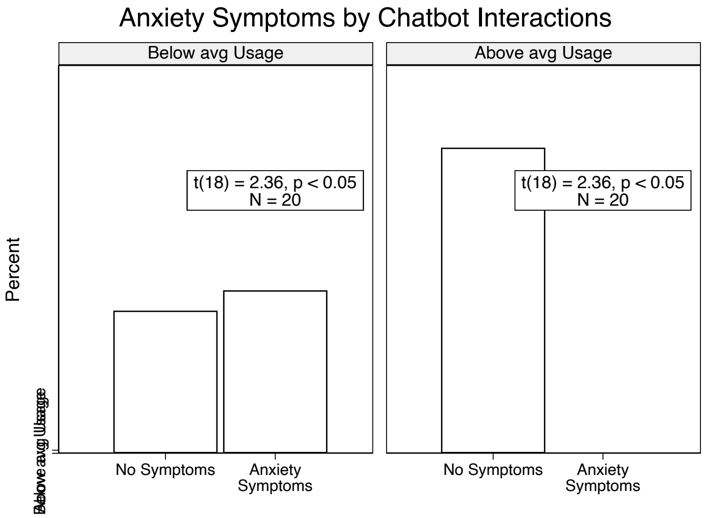

Figure S.18: Emotional openness to chatbot by mild anxiety levels.

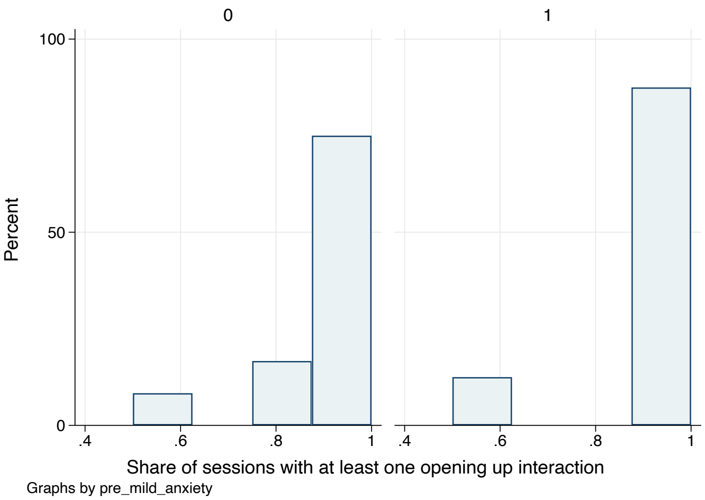

Figure S.19: Depressive symptoms by weekly user chatbot interaction

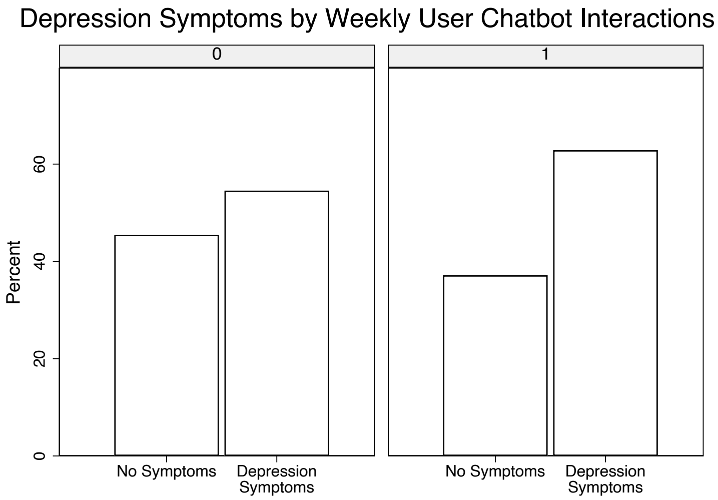

S.2. User Feedback on the Safespace App

This section presents qualitative feedback collected from participants regarding their experience using the Safespace app. The responses are divided into two parts: (1) direct feedback collected via Qualtrics surveys and (2) feedback recorded by university counselors during follow-up discussions. Translated responses from German are indicated as "(translated)", original text available upon request.

S.2.1. Feedback from Qualtrics Surveys

- "It was incredibly laggy to the point of being unusable."
- "Sometimes the chatbot gives confusing answers with brackets, slashes, and unusual words. Sometimes even with Russian letters. This is usually resolved by responding with a question mark, but it might still be worth looking into."
- "For me, it felt weird opening up to a chatbot. I much rather would talk to a friend face-to-face, but that's just my personal preference."

S.2.2. Feedback from Follow-Up Interviews with University Counselors

- (translated) "I used the app and was very impressed by it."
- (translated) "For the conversations I had with the app, I didn't feel the need for additional coaching. I used it to write down my thoughts and put them into words. It was very helpful to receive responses that either confirmed my thinking or provided a suggestion for reflection. I can imagine that using the app alongside coaching could be useful, but I don't think it is necessary."
- (translated) "Of course, I can imagine using the app before or after a session."
- (translated) "I talked about the same or similar topics with the chatbot as I did in my counseling sessions."
- (translated) "I ultimately didn't try the app because my questions had already been answered well elsewhere."
- (translated) "I preferred seeking advice and support from people rather than a chatbot. I have a strong network of friends and family, so I simply didn't feel the need to use it."
- (translated) "I regret to inform you that I ultimately did not use the app. I was uncertain about data security, so I decided against using it."
- (translated) "I ended up not using the app at all because it seemed like too much effort to fill out the mood tracker and other components daily. I have tried using similar tools in the past but could never stick with them, so I decided to forgo it entirely. Additionally, I was somewhat skeptical about an AI providing mental health advice—it felt too impersonal for something so personal, and I couldn't trust it."
- (translated) "I did not use the app."
- (translated) "Access did not work. I sent the flyer again."

- (translated) "The chatbot never ends the conversation. It would be useful if it could close a session itself; otherwise, there is a risk of distraction. Also, it frequently steers the conversation towards 'How are you?' even when that is not the topic I initiated."
- "I struggled to install the app because I had never used TestFlight before. My access code initially did not work, but the issue was resolved."
- (translated) "The app responds empathically, encouragingly, reassuringly, constructively, and appropriately. It was beneficial. The journal is good. A daily push notification to remind users to fill out the journal would be helpful. Having emergency contact numbers always available is useful. I would recommend the app to others."
- (translated) "I used the app 1-2 times per week. Topics included sleep issues, motivation, and difficulties with interpersonal relationships. The app provided detailed and comprehensive answers, responding with empathy and understanding. It always took context into account and suggested solutions within the conversation. I would like to continue using the app. It would be great if users could personalize the appearance of the app (e.g., colors, background). A daily push notification with an encouraging message related to discussed topics would be motivating."
- (translated) "I ended up not using the app because I forgot about it."
- "I used the app for a short time. I found the AI interaction realistic and motivating. However, after my initial curiosity, I didn't find the app personally useful anymore."
- (translated) "The chatbot usually repeats what you already know. It doesn't provide new insights, and the 'aha' moment is missing. The depth is lacking."
- (translated) "The installation process was complicated with various codes and the nested app-in-app setup. Once installed, though, the interface was simple to use."
- (translated) "The chatbot gave precise answers and generally found the right words. The responses were very rational, which I personally liked as I value efficiency. However, I imagine that if the chatbot is meant to simulate talking to a person, it would be better if it provided more detailed responses and asked follow-up questions—so that it feels more like a conversation rather than a back-and-forth Q&A."
- (translated) "I would like to continue using the app. It feels like chatting with a real person, but I don't see it as a replacement for coaching—rather, it's a good in-between option."
- (translated) "A daily push notification with an uplifting message related to what I've discussed in the chatbot would be motivating."

S.3. Survey Pre-Intervention

Consent form

This is a research project from ETH Zurich on AI and mental wellbeing. It is your choice whether or not to participate in this research. If you choose to participate, you may change your mind and leave the study at any time. Refusal to participate or stopping your participation will involve no penalty. This research question has been approved by the ETH Ethics Committee (EK 2023-N-184).

What can I expect if I take part in this research?

The study will last one month at most. The initial survey will take around 5 minutes to complete. A follow-up survey will be sent to you at the end of the study, and will take around 5 minutes to complete. At the end of the initial survey, if eligible, you will receive detailed instructions on how to access the mobile application "Safespace", that includes mood tracking and AI chatbot features. You may use the app as frequently as you like.

Once you begin a survey, you will not be able to leave it and return to it at another time, so please complete it in one sitting. There is also no "Back" button, so you cannot change responses once you proceed to the next page. Data from respondents will only be studied in an aggregated way. At the end of the survey you will receive a link to download the app and a unique identifier, with which you will be able to login in the application. At the end of the study period you will use the unique identifier to fill in the concluding survey.

What are the risks and possible discomforts?

If you choose to participate, answering survey questions that require reflection on issues related to your mental health and potentially distressing past experiences has some psychological risk. The information collected will not be used by the researchers to make a medical diagnosis. If you become upset or feel any distress when you are responding to these questions, please check your country's mental health services or contact medical support.

If I take part in this research, how will my privacy be protected? What happens to the information you collect in the survey and the application?

The data we collect will be stored on a secure server and analysed in an anonymous way. No raw, individual response-level background data will ever be made public. Such data will also not be handled or accessed by anyone other than research assistants hired by the researchers and the researchers. No attempt will ever be made to identify whether or how specific individuals answered the questions in this study. The ID provided to you for access to each survey cannot be connected to you and is intended to ensure that you only complete each survey once and to allow the researchers to connect the survey responses to the application usage. Data from the application are stored on a separate secure server from the dataset with your survey responses. The data collected by the app are minimal and include mood tracking, number of accesses, number and time of inputs. Data from iOS and Android testing platforms are discarded at the end of the study, they are not linked to the survey results or to usage inside the app. The content of your conversations with the chatbot is private, the content is stored locally on your phone and cannot be accessed by the researchers in any way. It will be automatically deleted from your device when you delete the app.

If I have any questions, concerns, or complaints about this research study, who can I talk to?

The lead researcher for this study is Matteo Pinna, who can be reached at IFW D33.3; help@safespaceresearch.com. Please contact him if you have questions, concerns, complaints, or If you wish to notify explicitly your withdrawal from the study.

Do you consent to participate in this study?

- ◯ By clicking this box, I confirm that I have read and I understand the information above and consent to participate.
- ◯ No

Wave num

2

Students

Are you a student from:

- ◯ ETH
- ◯ UZH
- ◯ Other institution
- ◯ Not a student

Are you a:

- ◯ Bachelor student
- ◯ Master student
- ◯ PhD student
- ◯ Other

Where you directed to this study by the ETH Student Counselling? (You may participate either way)

- ◯ Yes
- ◯ No

Who was the counsellor you had a session with? (name and last name if possible)

Background

How old are you?

What country do you live in?

What is your country of primary citizenship?

Are you a second generation immigrant?

- ◯ Yes
- ◯ No

Which of the following races best describe(s) you: (Select all that apply)

- ☐ American Indian or Alaska Native
- ☐ South Asian (for example, Indian, Pakistani, Sri Lankan, etc.)
- ☐ East Asian (for example, Chinese, Japanese, Korean, etc.)
- ☐ Southeast Asian (for example, Vietnamese, Filipino, Indonesian, etc.)
- ☐ Middle Eastern or North African
- ☐ Pacific Islander
- ☐ Indigenous or Aboriginal
- ☐ Black or African
- ☐ White
- ☐ Other

Which of the following languages are you fluent in? (Select all that apply)

- ☐ Swiss German
- ☐ High German
- ☐ English

- [ ] French
- [ ] Italian
- [ ] Other

Which of the following describes your gender identity?

- ◯ Male
- ◯ Female
- ◯ Transgender
- ◯ Non-binary
- ◯ If not listed above, please write your gender identity here.

How would you best describe your current relationship status?

- ◯ Single
- ◯ Casual
- ◯ Dating
- ◯ Long-term/Committed
- ◯ Married
- ◯ Divorced
- ◯ Widowed
- ◯ Other

Do you live alone?

- ◯ Yes
- ◯ No

Safespace

Please select to what extent you agree with the statement: "The Safespace application's AI chatbot will provide me with helpful advice regarding mental health".

- ◯ Strongly agree
- ◯ Somewhat agree
- ◯ Neither agree nor disagree
- ◯ Somewhat disagree

◯ Strongly disagree

I trust that my privacy will be well-protected in the Safespace application.

◯ Strongly agree
◯ Somewhat agree
◯ Neither agree nor disagree
◯ Somewhat disagree
◯ Strongly disagree

Mental health

How would you rate your mental health overall?

◯ Poor
◯ Fair
◯ Good
◯ Excellent

About how many people do you have in your personal life that you can really open up to about your most private feelings without having to hold back?

	0	1	2	3	4	5	6	7	8	9	10	More than 10
Number												☐

Over the last two weeks, how often have you been bothered by any of the following problems?

	Not at all	Several days	More than half the days	Nearly every day
Little interest or pleasure in doing things	◯	◯	◯	◯
Feeling down, depressed, or hopeless	◯	◯	◯	◯
Trouble falling or staying asleep, or sleeping too much	◯	◯	◯	◯
Feeling tired or having little energy	◯	◯	◯	◯
Poor appetite or overeating	◯	◯	◯	◯

	Not at all	Several days	More than half the days	Nearly every day
Feeling bad about yourself — or that you are a failure or have let yourself or your family down	○	○	○	○
Trouble concentrating on things, such as reading the newspaper or watching television	○	○	○	○
Moving or speaking so slowly that other people have noticed? Or the opposite — being so fidgety or restless that you have been moving around a lot more than usual	○	○	○	○
Thoughts of not wanting to be alive or causing harm to oneself	○	○	○	○

Over the past two weeks, how often have you been bothered by the following feelings?

	Not at all	Several days	More than half the days	Nearly every day
Feeling nervous, anxious or on edge	○	○	○	○
Not being able to stop or control worrying	○	○	○	○
Worrying too much about different things	○	○	○	○
Trouble relaxing	○	○	○	○
Being so restless that it is hard to sit still	○	○	○	○
Becoming easily annoyed or irritable	○	○	○	○
Being afraid as if something awful might happen	○	○	○	○

Unfortunately we cannot offer you access to the Safespace application at the moment. If you need further information, please contact Matteo Pinna at matteo.pinna@gess.ethz.ch.
We thank you for your time spent taking this survey. Your response has been recorded.

randomID

This is your unique, 8-digit anonymous ID:

${e://Field/uniqueID}

Please refrain from sharing this ID with anyone else. For your inputs not to go to waste, it is essential that you keep this unique ID safe. If you lose or forget this ID, you will not be able to login to the application for the study.

Please take a moment now to write down, or copy your unique ID and check the paste function is working correctly. Have you noted your unique ID safely?

○ No
○ Yes

Application download

What is your smartphone's operative system?

○ iOS (Iphone)
○ Android (Samsung, Google, Oppo, Honor etc.)
○ Other

You will now be provided a link. The link will ask you to install TestFlight, Apple's testing platform. After installing TestFlight, you will be able to download Safespace from it. Please click on the following link to install Safespace on iOS: https://testflight.apple.com/join/WAomJmq7. In Step 1, access the app store and download TestFlight, then click on Start Testing in Step 2. Be sure to have copied the link or installed the app, without losing the unique ID, before leaving this page. You will have to insert the unique ID within the Safespace application, NOT in the the TestFlight interface.
If you have any issue please write us at help@safespaceresearch.com with your unique ID, we are very responsive.

In the next window you will now be provided an installation .apk file, please do not share it. Before going to the next window please allow for installations from unknown sources on your phone, without closing this survey's page:

1. **To enable installations from unknown sources, go to your device's settings. This is usually represented by a gear icon. Scroll down and tap on "Security" or "Security & Privacy".**
2. **Depending on your Android version, you might need to look for "Biometrics and security" or "Apps & notifications". Scroll down and find the "Install unknown apps" or "Unknown sources" option. Again, this could be slightly different depending on your Android version. Turn the toggle on or tap on the option to enable it.**
3. **You may get a warning message about the potential risks of installing unknown apps. Accept it to proceed.**
4. **Once you've done this, open the .APK installation file in the next window to install the App.**

If you have any issue please write us at help@safespaceresearch.com with your unique ID, we are very responsive.

Please download the installation .apk file at this link: https://safespacestorageaccount.blob.core.windows.net/apk/safespace-1.0.1.apk. Before exiting this page, please be sure to have downloaded the .apk on your device or saved the link.
If you have any issue please write us at help@safespaceresearch.com with your unique ID, we are very responsive.

S.4. Survey Post-Intervention

Consent form

Thank you for participating!

This is a research project from ETH Zurich (EK 2023-N-184). By choosing to complete this follow-up survey, you help us to collect complete and useful data for our research. Your participation in this follow up is not mandatory and you can drop out at any time.

What can I expect if I take part in this follow-up?

The follow-up survey will take around 5 minutes to complete. And involves similar questions to the ones you answered at the beginning of the study. Once you begin a survey, you will not be able to leave it and return to it at another time, so please complete it in one sitting. There is also no "Back" button, so you cannot change responses once you proceed to the next page. Data from respondents will only be studied in an aggregated way.

What are the risks and possible discomforts?

If you choose to participate, answering survey questions that require reflection on issues related to your mental health and potentially distressing past experiences has some psychological risk. The information collected will not be used by the researchers to make a medical diagnosis. If you become upset or feel any distress when you are responding to these questions, please check your university's mental health services or contact medical support. You can find information here on the ETH services. The Swiss help and emergency line is 112.

What happens to the information you collect in the follow-up survey?

The data we collect will be stored on a secure server and analysed in an anonymous way. No raw, individual response-level background data will ever be made public. Such data will also not be handled or accessed by anyone other than research assistants hired by the researchers and the researchers. No attempt will ever be made to identify whether or how specific individuals answered the questions in this study. The ID provided to you for access to each survey cannot be connected to you and is intended to ensure that you only complete each survey once and to allow the researchers to connect the survey respondents to the application usage.

If I have any questions, concerns, or complaints about this research study, who can I talk to?

The lead researcher for this study is Matteo Pinna, MPhil, who can be reached at IFW E46; matteo.pinna@gess.ethz.ch. Please contact him if you have questions, concerns, complaints, or If you wish to withdraw from the study.

What is your unique ID indicated on the application's screen?

Safespace

I used the Safespace app when I needed emotional or mental health-related advice.

- ◯ Always
- ◯ Most of the time
- ◯ About half the time

- Sometimes
- Never

I could freely share my emotional or mental health issues with the Safespace app.

- Strongly agree
- Somewhat agree
- Neither agree nor disagree
- Somewhat disagree
- Strongly disagree

The Safespace application's AI chatbot has provided me with helpful advice regarding mental health.

- Strongly agree
- Somewhat agree
- Neither agree nor disagree
- Somewhat disagree
- Strongly disagree

I usually felt better after chatting with the Safespace app.

- Strongly agree
- Somewhat agree
- Neither agree nor disagree
- Somewhat disagree
- Strongly disagree

I trust that my privacy is be well-protected in the Safespace application.

- Strongly agree
- Somewhat agree
- Neither agree nor disagree
- Somewhat disagree
- Strongly disagree

The Safespace app provided harmful advice.

- Strongly agree
- Somewhat agree
- Neither agree nor disagree
- Somewhat disagree
- Strongly disagree

The Safespace app was easy to use.

- Strongly agree
- Somewhat agree
- Neither agree nor disagree
- Somewhat disagree
- Strongly disagree

I would like to continue using the Safespace app even after the completion of this study.

- Strongly agree
- Somewhat agree
- Neither agree nor disagree
- Somewhat disagree
- Strongly disagree

I have used the recording/play audio features of the chatbot

- Always
- Most of the time
- About half the time
- Sometimes
- Never

Is there anything else that you want to tell us about your experience with the Safespace app? Any feedback can help us to improve!

Mental health

How many times have you received professional counselling during the study period? (e.g. ETH Counselling Service, Psychotherapy)

	0	1	2	3	4	5	6	7	8	9	10	More than 10
Number												☐

Over the last two weeks, how often have you been bothered by any of the following problems?

	Not at all	Several days	More than half the days	Nearly every day
Little interest or pleasure in doing things	○	○	○	○
Feeling down, depressed, or hopeless	○	○	○	○
Trouble falling or staying asleep, or sleeping too much	○	○	○	○
Feeling tired or having little energy	○	○	○	○
Poor appetite or overeating	○	○	○	○
Feeling bad about yourself — or that you are a failure or have let yourself or your family down	○	○	○	○
Trouble concentrating on things, such as reading the newspaper or watching television	○	○	○	○
Moving or speaking so slowly that other people have noticed? Or the opposite — being so fidgety or restless that you have been moving around a lot more than usual	○	○	○	○
Thoughts of not wanting to be alive or causing harm to oneself	○	○	○	○

Over the past two weeks, how often have you been bothered by the following feelings?

	Not at all	Several days	More than half the days	Nearly every day
Feeling nervous, anxious or on edge	○	○	○	○
Not being able to stop or control worrying	○	○	○	○
Worrying too much about different things	○	○	○	○
Trouble relaxing	○	○	○	○
Being so restless that it is hard to sit still	○	○	○	○

	Not at all	Several days	More than half the days	Nearly every day
Becoming easily annoyed or irritable	◯	◯	◯	◯
Being afraid as if something awful might happen	◯	◯	◯	◯
